# Partial least Squares- least squares- Support Vector Machine Modeling of ATR-IR as a Spectrophotometric Method for Detection and Determination of Iron in Pharmaceutical Formulations

**Published:** 2019

**Authors:** Elahehnaz Parhizkar, Hadi Saeedzadeh, Fatemeh Ahmadi, Mohammad Ghazali, Amirhossein Sakhteman

**Affiliations:** a ***Department of Pharmaceutics, School of pharmacy, Shiraz university of medical sciences, Shiraz, Iran.***; b ***Department of Medicinal chemistry, School of pharmacy, Shiraz university of medical sciences, Shiraz, Iran.***; c ***Medicinal and natural products chemistry research center, School of Pharmacy Shiraz University of Medical Sciences, Shiraz, Iran.***

**Keywords:** Attenuate Total Reflectance Mid-infrared, Atomic absorption spectroscopy, Iron, Partial least squares- least squares- support vector machine model

## Abstract

Iron is an essential element used as supplement in different dosage-forms. Different time and expenditure-consuming methods introduced for detection and determination of elemental ions such as Atomic Absorption Spectroscopy. In this research, two different and routine methods containing ATR-IR and atomic absorption were applied to define the amount of iron in 198 samples containing different concentrations of commercial iron drops and syrups and the output data of the methods was transferred to chemometric model to compare the accuracy and robustness of the methods. By applying this mathematical model, in addition to the confirmation of ATR-IR (a time and energy-saving method) as a replacement of Atomic Absorption Spectroscopy to produce the same results, chemometrical model was used to evaluate the output data in a faster and easier method. At first, ATR-IR spectra data converted to normal matrix by SNV preprocessing approach. Then, a relationship between iron concentrations achieved by AAS and ATR-IR data was established using PLS-LS-SVM. Consequently, model was able to predict ~99% of the samples with low error-values (root mean square-error of cross-validation equal to 0.98). Y-permutation test performed to confirm that the model was not assessed accidentally. Although, chemometric methods for detection of some heavy metals have been reported in the literature, combination of PLS-LS-SVM with ATR-IR was not cited. In this study a fast and robust method for iron assay was suggested.As a result, ATR-IR can be a suitable method in detection and qualification of iron-content in pharmaceutical dosage forms with less energy-consumption but similar accuracy.

## Introduction

Inorganic nutrients are essential in diet due to the vital roles playing in metabolism, growth, and development of human being. These compounds are provided as food nutrition or as supplements (1-3). One of the most important inorganic supplements is iron due to its role in metabolic implements such as myoglobin, hemoglobin, and proteins such as cytochromes. Lack of Iron, which is called iron deficiency anemia, causes symptoms including light-headedness, breathlessness, weakness, fatigue, faintness, and cold sensitivity (2, 4-7). 

According to iron roles, it is recommended to be consumed as supplements in all ages (2, 4, 8, 9). Many commercial products of Iron in different dosage forms such as oral drop, syrups, capsules, tablets, and injections are available in drug market. However, the important difference between these products is the amount of elemental iron, the absorbable form of iron in the body (2). Therefore, many physicians recommend a special pharmaceutical brand to be administered and believed in higher effectiveness of the drug because of higher elemental iron amount. Therefore, establishing a simple and yet functional method to determine the iron content in pharmaceutical products is essential. 

Many different electrochemical and chromatographic methods are employed in metal determination such as atomic absorption spectroscopy, inductively coupled plasma optical emission spectrometry, spectrofluorimetry, electrophoresis, voltammetry, spectrophotometry, and chromatography (3, 4, 6, 10-12). 

In most of the mentioned methods, sample preparation is a time consuming method that needs the use of expensive blank materials and solvents (3, 4, 11, 13).

In recent years, many attempts have compassed the use of spectrophotometry-based techniques, because of their rapidity, simplicity, and less material consuming processes (10, 12, 14). 

ATR (Attenuate Total Reflectance) Mid-infrared (MIR) spectroscopy was shown to be a non-invasive and facile technique in drug and food analysis and control (15-18). This technique introduces advantages such as high speed performance, no need for reagents, ability for detection of a special component in complex matrix and analysis without sample destruction (4, 19-21).

In IR spectroscopy of complex systems, the absorbance bands are highly overlapped. Therefore, the output data have to be analyzed and interpreted by chemometric tools for dispelling this disadvantage (12, 21).

A well-known method for detection of metal elements in pharmaceutical samples is atomic absorption spectroscopy (AAS). Although AAS is reproducible and easy to use, the sample is (being) should be omitted destroyed during analysis. Meanwhile, sample preparation and stabilization of the instrument is time consuming. Other disadvantages of AAS include its expensiveness and presence of interference in analysis (13, 22). The aim of this paper is to present an alternative non-invasive method for AAS based on analysis of ATR-IR data in order to determine iron in pharmaceutical products. Partial least squares- least squares- support vector machines (PLS-LS-SVM) as a nonlinear chemometric approach, have been applied for mathematical modeling. 

Correlation of ATR-IR and AAS can be a leading point in presenting a novel assay method for detection of iron in pharmaceutical preparations.

In some previous studies (4, 10, 12), applying PLS chemometrics model, the amount of some heavy metals were determined in different samples by spectrophotometric or voltammetry methods. However, combination of ATR-IR and AAS, with a model based on a hybrid model of PLS and SVM was not reported previously. 

## Experimental

*Reagents and software*

All the materials used in this research were of analytical grades purchased from Merck (Darmstadt, Germany). Modeling calculations were performed on a Core™i5 (Intel) laptop employing Matlab® software (Mathworks Inc., Natick, MA, USA) with the SMV Toolbox (Eigenvesctor Research Inc., version R2012a). Extra manipulations on spectra, referred in the following sections, were operated with our own codes written in Matlab® software package.

*Reference treatment*

A reference solution was prepared by diluting appropriate amounts of Fe^++^, as reference material of flame atomic absorption spectroscopy (AAS) (varian spectra. 20 PLUS, USA), to calibrate the instrument for iron assay. Output data was analyzed to set a calibration of elemental iron concentrations against absorbance. 

*Sample preparation*

An aqueous solution of standard solutions of marketed ferrous sulphate drops and syrups were prepared. Accordingly, the concentration of iron in the samples was within the range of 0.5- 25 mg L^-1^. Linear correlation between iron concentration and AAS data were explored. Each company contains three different batches and all samples were studied tree times. The range of selected concentrations was chosen due to common concentrations in pharmaceutical assays. 

*Sample preparation for ATR-IR analysis*

All samples prepared by the previous method were subjected to ATR-IR spectroscopy and the absorbance data were studied by ATR-IR spectrophotometer (Bruker, VERTEX 70, Germany). Spectra were recorded by a diamond crystal prism equipped cell between 4000 cm^−1^ and 400 cm^-1^ with nominal resolution 4 cm^−1^ and 128 co-added scans. Distilled water was consumed as reference for the background spectrum before collecting samples spectra (n = 3).

*Chemometrics model proportion*

*ATR-IR data preprocessing*

The ATR-IR spectra of iron samples in different concentrations were processed by standard normal variate method (SNV method) to improve the resolution of overlapping bands and reduce baseline offsets. Furthermore, by this method, AAS data are related to ATR-IR wavenumber intensity (23, 24).

*Data separation*

ATR-IR outputs were divided into calibration (training) and validation (test) sets by applying Kennard-stone algorithm. This algorithm is based on Euclidean distance of data set that means; the first two objects are selected in a manner to have maximum distance from each other. The third object is selected in a way to have the farthest distance from the primarily selected set (25-27). 158 samples were used for setting a calibration set and 40 samples were used as test set. 

*Model selection and validation*

The obtained calibration matrix from Kennard-stone algorithm was subjected to a m-file for performing PLS-LS-SVM calculations on data set. Gaussian-RBF was used as the function type in PLS-LS-SVM calculations. Predicted residual error sum of squares (PRESS) inside calibration set was used as an error metric to select the optimum number of latent variables for further validations. Model robustness was determined by leave-one-out (LOO) and leave-many-out (20%) (LMO) cross validation methods. These methods are based on excluding the samples from the data set and estimating the excluded values based on the obtained model (26, 28). This process is done for all samples to show the stability of the model. 

External validation analysis was employed to predict the responses in the test set based on calibration data set. PRESS and R^2^ of external validation show the predictive ability of the models against external data sets. 

*Predictability of the model*

Response permutation test was operated to assure that the model was not obtained accidentally. For this reason, randomly ordered response vectors (Y) (AAS data) were assigned several times to establish calibration set. Obtaining high error values by scrambled vectors means that the presented model was not achieved randomly. 

## Results and Discussion

*AAS method data*

**Figure 1:**
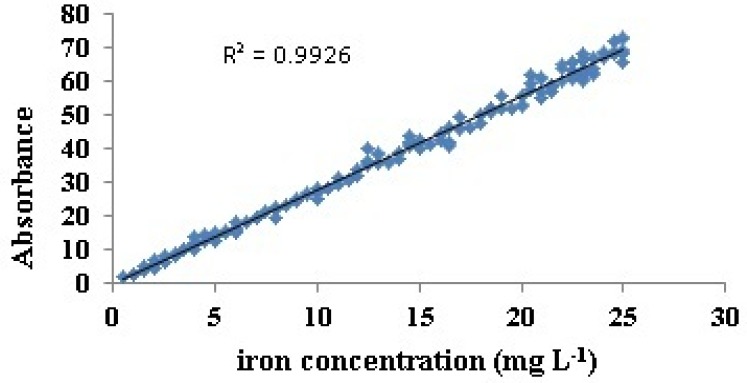
Linear correlation between iron concentrations and AAS absorbance data

AAS absorbance output was plotted against corresponding concentrations that showed linear relation (R^2 ^= 0.99) (Figure 1). It was therefore verified that the selected samples in this study were within the range of Beer-Lambert law based on AAS method. This result is a proof of the ability of AAS method to determine the iron in linear and reproducibility.

*Setting a multivariate model between ATR-IR and AAS values*

A multivariate model between spectral data of ATR-IR and AAS was the main purpose of this research. On this way, PLS-LS-SVM artificial intelligence method was used in such a way that AAS data were the response vector (Y values) and ATR-IR data at different wavenumbers were considered as X matrix.

**Table 1:** PLS-SVM models for calibration set of ATR-IR data (x) and AAS (y).

	**NO. of LVs**	**1**	**2**	**3**
Prediction	r^2^	0.536	0.992	0.994
	PRESS	8.685	0.1417	0.112
Cross Validation (LOO)	Q^2^	0.472	0.976	0.984
	PRESS	9.81	0.44	0.28
Cross Validation (LMO)	Q^2^	0.502	0.912	0.979
	PRESS	8.121	0.432	0.325

**Table 2:** Y-permutation test for approving PLS-LS-SVM model proficiency

**Metric**	**value**	
R^2^ external validation	0.95	External Validation
PRESS external validation	0.3	
R^2^ Y-permutation test	<0.22	Chance Correlation
R^2^ Y-permutation test LOO	<0.28	
R^2^ Y-permutation test LMO	<0.31	

**Figure 2:**
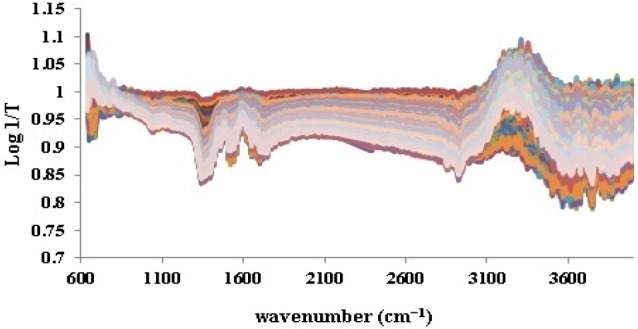
ATR-IR spectra of 198 iron sulphate samples

**Figure 3:**
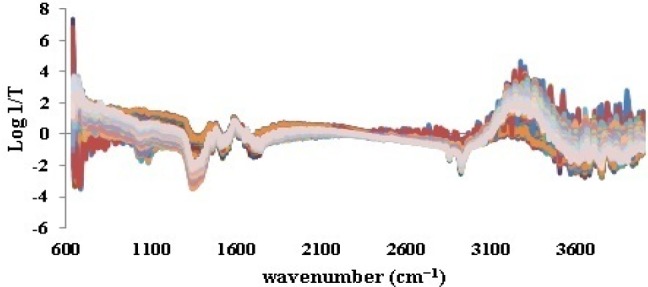
SNV processing of ATR-IR spectra of iron samples

**Figure 4:**
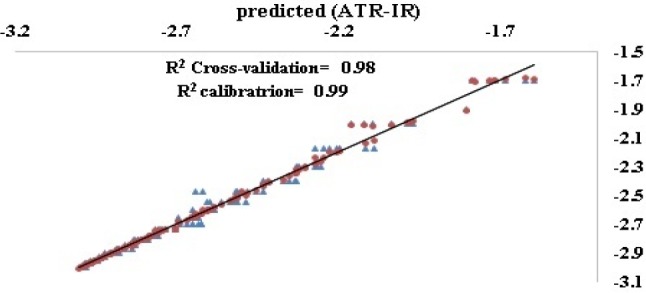
Experimental values (AAS) vs. Predicted values (ATR-IR) for prediction (●) and test (▲) sets

This modeling approach, using quadratic programming, was proposed by Vapnik in 1988 in statistical field to solve convex optimization queries. In progress, Suykens and Vandewalle offered more simple linear equations called least squares support vector machine (LS-SVM) that use less number of coefficients and causes better performances (12, 14). 

Partial least squares regression (PLS) is a modeling technique based on linear relationship between the analytical signals and the special considered property (12).

As it was noticed previously, the ATR-IR spectra of all 198 iron samples (Figure 2) were divided into 158 calibration and 40 validation test data by applying Kennard-stone method. Prior to splitting, base line correction was fulfilled by means of SNV (Figure 3). This step was done to disallow fluctuations of base line in different samples to be considered as signals. 

The SNV method obeys the following equation (29):

SNVi=Xi-X®∑i(Xi-X)®2n-12

X_i _is ATR-IR intensity at each specific wavenumber, X is ATR-IR mean strength and n as number of wavenumbers. The response vector was considered as the p-function of AAS results.

In order to generate a matrix at lower dimensions, Non-linear iterative partial least squared (NIPALS) based PLS was used to avoid presence of noise in the input matrix of LS-SVM. As mentioned in material section, error metrics of calibration, LOO and LMO cross validation sets were considered as the principal metrics for optimizing number of latent variables in the final model. As seen in Table 1, three latent variables resulted in a model with root mean square error of calibration (R^2^) 0.99 and that of cross validation (Q^2^) 0.98 (Figure 4). 

The plot also reveals that no overfitting is observed in case of this assay method, since a pitfall of artificial methods is that they are susceptible to overfitting. In this case the model is adapted to input data but it is not able to predict the external data. Using different validation techniques and external data, it was verified that the model was not over fitted in this study. Therefore, the concluded PLS-LS-SVM model was statistically qualified (Table 1) in a way to predict about 99% of variables.

*Model validation*

Machine learning methods such as PLS-LS-SVM model are highly prone to overtraining (26). Therefore, it was very significant to validate the obtained model. 

As illustrated in Table 1, the combination of 2 and 3 PLS latent variables (LVs) with support vector machines resulted in appropriate models. By applying one latent variable, R^2^ values of prediction set, Q^2 ^of leave-one-out, and leave-many-out cross validation were not within acceptable range. This means that the model with one latent variable was highly under the influence of overtraining. On the other hand, in case of 2 and 3 latent variables reasonable R^2^ values for prediction were achieved. Robustness of the model in case of 3 latent variables was at most (LOO = 0.98, LMO = 0.97) presenting it as the most reasonable model in this study. The model with 3 variables was therefore selected as the most appropriate model to be used for prediction of test data set. As seen in Table 2, predictive ability of the final model was also validated (R^2^ external validation = 0.95, Press = 0.3).

*Model predictability*

To certify that the 3 latent variable based model was not achieved accidentally, Y-permutation test was performed for training set and LOO/ LMO cross validation. Accordingly, randomly unsorted AAS vectors (Y) were applied many times to instruct calibration data set (30). The resulted high error values of the random models output enforced that the resulted model was not obtained by chance (Table 2).

## Conclusion

An ATR-IR method was introduced to determine iron in commercial drop and syrups. This technique can be an efficient alternative assay method for AAS in iron determination. The method needs less expenditure, time and materials and can be operated more easily. The use of chemometric model PLS-LS-SVM with 3 latent variables allowed optimum prediction for iron sulphate concentration. 

In this study, various concentrations of ferrous pharmaceutical samples were prepared and their concentrations were evaluated by two different methods, AAS, and ATR-IR. The output data were used as input data of chemometric model to compare the detection ability of the iron in two mentioned methods. AAS and ATR-IR spectral data were compared and the similarity of both methods in detection and qualification of iron was affirmed. The model validity was tested by permutation test and showed low correlations for random data meaning that model responses were not achieved accidentally. The best correlation was determined by applying PLS-LS-SVM as a proper chemometrics model which can show the best fitness of the data and the model.

This study ensures that the iron potency value obtained by simple method ATR-IR is similar to routine analysis (AAS) and can be considered as a sturdy replacement in pharmaceutical control assays.
